# Phase-Change Microcapsules with a Stable Polyurethane Shell through the Direct Crosslinking of Cellulose Nanocrystals with Polyisocyanate at the Oil/Water Interface of Pickering Emulsion

**DOI:** 10.3390/ma16010029

**Published:** 2022-12-21

**Authors:** Denis Voronin, Rais Mendgaziev, Adeliya Sayfutdinova, Maria Kugai, Maria Rubtsova, Kirill Cherednichenko, Dmitry Shchukin, Vladimir Vinokurov

**Affiliations:** 1 Department of Physical and Colloid Chemistry, National University of Oil and Gas ”Gubkin University”, 119991 Moscow, Russia; 2 Department of Chemistry, Stephenson Institute for Renewable Energy, University of Liverpool, Liverpool L69 7ZD, UK

**Keywords:** phase-change materials, *n*-octadecane, latent heat storage, nanocrystalline cellulose, interfacial polymerization, branched oligo-polyol, rigid polyurethane

## Abstract

Phase-change materials (PCMs) attract much attention with regard to their capability of mitigating fossil fuel-based heating in in-building applications, due to the responsive accumulation and release of thermal energy as a latent heat of reversible phase transitions. Organic PCMs possess high latent heat storage capacity and thermal reliability. However, bare PCMs suffer from leakages in the liquid form. Here, we demonstrate a reliable approach to improve the shape stability of organic PCM *n*-octadecane by encapsulation via interfacial polymerization at an oil/water interface of Pickering emulsion. Cellulose nanocrystals are employed as emulsion stabilizers and branched oligo-polyol with high functionality to crosslink the polyurethane shell in reaction with polyisocyanate dissolved in the oil core. This gives rise to a rigid polyurethane structure with a high density of urethane groups. The formation of a polyurethane shell and successful encapsulation of *n*-octadecane is confirmed by FTIR spectroscopy, XRD analysis, and fluorescent confocal microscopy. Electron microscopy reveals the formation of non-aggregated capsules with an average size of 18.6 µm and a smooth uniform shell with the thickness of 450 nm. The capsules demonstrate a latent heat storage capacity of 79 J/g, while the encapsulation of *n*-octadecane greatly improves its shape and thermal stability compared with bulk paraffin.

## 1. Introduction

Phase-change materials (PCMs) are actively studied regarding their capability of storing and releasing thermal energy as latent heat of reversible phase transitions [1]. PCMs are considered as emerging additives to bring responsive thermoregulation and enhanced thermal functionality in many valuable technologies, such as space conditioning and heating [2,3], smart textiles [4,5], photovoltaics [6,7], hand-held electronics [8] and lithium-ion batteries [9,10]. For instance, according to International Energy Agency (IEA), in 2021, half of the energy demanded in buildings was used for space and water heating [11]. The majority of heating energy demands are still meet by fossil fuels, which produce about 2450 Mt of direct CO_2_ emissions [12]. The employment of PCMs in in-building thermal regulation will allow us to accumulate excess thermal energy at times of peak supply, including from renewable sources, and release it in response to environmental changes to improve indoor comfort and decrease energy requirements [13,14]. Additionally, heat is the most common form of energy loss. Therefore, the development of reliable and efficient technologies for thermal energy storage is essential for rational energy consumption and recycling.

A significant advantage of latent heat storage with PCMs is a higher density of stored energy in a narrower temperature range, compared with conventional sensible heat storage [3]. Organic PCMs, such as paraffins and fatty acids, can store and release a large amount of latent heat during the melting and crystallization of long methylene chains with a wide range of phase transition temperatures and durability of thermal properties [15,16]. However, organic PCMs are hardly applicable as thermoregulating additives in the pristine form, as they suffer from leakage in the melted state. To address the leakage issue, PCMs may be either encapsulated by sealing into micro- and nanocontainers with a tough shell, or adsorbed onto the supporting materials with a high specific surface area [17,18,19]. Encapsulated PCMs strongly benefit from improved shape stability and thermal reliability, enhanced heat transfer area and prevention of their reactivity with the surrounding medium [20,21].

Chemical encapsulation via interfacial polymerization was shown to be a versatile approach to encapsulating organic PCMs [22,23]. As a rule of thumb, encapsulation via interfacial polymerization implies the preparation of an oil-in-water emulsion stabilized with an appropriate surfactant, with hydrophobic monomers dispersed in the oil phase. The encapsulation takes place when the hydrophilic monomer is added to the aqueous phase and polymerization is initiated by adjusting the pH or mixture temperature. The monomers react at the oil/water interface of the emulsion droplet, and entrap the PCM dissolved in the oil core into the resulting polymeric shell.

In this way, organic PCMs encapsulated into a polyurethane (PU) shell were prepared [24,25]. The PU shell is formed via interfacial polycondensation (a particular type of polymerization) as a result of a reaction between isocyanates dissolved in the oil phase and polyols dissolved in the aqueous phase, respectively. The resulting PU shell appears as a matrix of soft polymeric chains reinforced with highly crosslinked hard domains of urethane groups [26]. This allows for adjusting the mechanical properties of the shell by matching isocyanates and polyols of various structures and functionalities [27]. Additionally, PUs are insoluble in most common solvents, which makes the PCM loaded PU capsules perfect candidates to be thermoregulating additives in different construction materials, like cement-based finishing layers or paints [28].

The formation of a PU shell via conventional interfacial polymerization requires an emulsifier to stabilize the oil/water interface, and a polyol as a source of hydroxyl groups to crosslink the shell with polyurethane linkages through reaction with isocyanate. In this work, we propose the encapsulation of organic PCM *n*-octadecane by the formation of a Pickering emulsion stabilized with nanocrystalline cellulose (NCC). The stable PU shell was achieved through the reaction of cellulose surface hydroxyl groups with polyisocyanate dispersed in the oil phase. Owing to its unique properties, such as large specific Young’s modulus, small size (100–200 nm in length and 5–10 nm in cross-section), high aspect ratio (15–30), and amphiphilicity, NCC appears as to be a promising reinforcing agent, rheological modifier and interface stabilizer [29]. Additionally, cellulose is a linear macromolecule composed of repeating rings of *β*-l,4-linked D-glucose units aggregated by numerous strong intermolecular hydrogen bonds [30]. In PU chemistry, D-glucose is considered one of the most important precursors in the synthesis of various polyolic starters for rigid polyether polyols, including *α*-methyl glucoside, hydroxyethyl glucoside and sorbitol [27]. On the other hand, a cellulose polymeric chain contains 6 hydroxyl groups per monomeric unit itself and, therefore, may potentially act as polyol in reaction with isocyanate.

To date, NCC has been used as a reinforcing agent in PU capsule shells [31], yet has never been employed as a participant in PU shell crosslinking, to the best of our knowledge. Here, we demonstrated the preparation of phase-change capsules via interfacial polymerization, employing NCC simultaneously as emulsion stabilizer and oligo-polyol to crosslink the PU shell. The resultant NCC/PU capsules were intact, non-aggregated and yet polydisperse, with a mean diameter of 18.6 µm. On top of that, the capsules revealed an adequate latent heat storage and release capability, with excellent shape stability and thermal reliability.

## 2. Materials and Methods

### 2.1. Materials

The commercially available polymeric diphenylmethane diisocyanate Wannate PM-200 (PAPI) with an average functionality of 2.6–2.7 was purchased via local supplier from Yantai Wanhua Polyurethanes (Yantai, China). *N*-octadecane paraffin (90%) was purchased from Alfa Aesar (Kandel, Germany). Nanocrystalline cellulose (NCC) was purchased from CelluForce (Montreal, QC, Canada). Nile Red dye, 4,N,N-Trimethylaniline (TMA, 99%), and toluene (≥99.5%) were purchased from Sigma-Aldrich (Darmstadt, Germany). All of the reagents were used without further purification.

Millipore Milli Q deionized (DI) water (18.2 MΩ·cm) was used in all sets of experiments requiring an aqueous medium, or preparation of aqueous solutions.

### 2.2. Synthesis of NCC/PU Capsules Loaded with n-Octadecane

The capsules were synthesized via polymerization on the oil/water interface of Pickering emulsion stabilized with NCC. At the first step, an oil-in-water Pickering emulsion was prepared. The water phase was 80 mL of 0.6 wt% NCC aqueous suspension. The oil phase consisted of 3.2 g of PAPI, 3.2 g of *n*-octadecane, and 300 µL of TMA dissolved in 18.5 mL of toluene. The emulsion was prepared by the pouring of oil and water phases, and following that, homogenization at 15,000 rpm for 2 min with a T 18 Ultra-Turrax disperser (IKA). The prepared emulsion was transferred to a two-neck round-bottomed flask equipped with a thermometer and a reflux condenser. The mixture was then heated to 70 °C under gentle stirring within 10 min. The reaction was complete after an additional 2 h of mixing at 70 °C. The resulting capsules were decanted, washed for five times with DI water and dried at room temperature in the desiccator.

### 2.3. Characterization of the NCC/PU Capsules

The surface morphology and shell thickness of the PU capsules were studied with an SEM-FIB JIB-4501 (JEOL, Akishima/Tokyo, Japan) electron microscope at an accelerating voltage of 5 kV. To find out the shell thickness, at least 15 SEM images of the broken capsules were captured and further processed with ImageJ software. Additionally, the capsule structure was investigated with a JEOL JEM 2100 UHR transmission electron microscope (TEM) at an acceleration voltage of 200 kV. The sample powders were dispersed in DI water and dropped onto a Lacey formvar/carbon TEM grid (Ted Pella, Inc., Redding, CA, USA). The samples were treated in a HPT-100 plasma cleaner (Henniker Plasma, Runcorn, UK) prior to insertion into the microscope chamber, in order to remove organic residues from the grid surface. The acquisition of micrographs was performed with the help of an Quemesa 11 MegaPixel CCD (Olympus, Shinjuku/Tokyo, Japan) camera in TEM mode.

The formation of a capsule shell and encapsulation of *n*-octadecane was studied with a Nicolet iS10 (Thermo Scientific, Waltham, MA, USA) FTIR spectrometer with germanium ATR crystal. The absorption spectra were collected in the 4000–600 cm^−1^ range as an averaged result of 16 iterative scans.

The crystallinity of NCC, *n*-octadecane, and NCC/PU capsules was examined with a MiniFlex 300/600+ (Rigaku, Akishima/Tokyo, Japan) X-ray diffractometer (40 kV, 15 mA) with Cu Kα radiation (λ = 0.154 nm). The diffraction patterns were collected at room temperature. The Bragg angles were between 5° and 50°, and the scanning rate was 3°/min.

The formation of the PU shell was also followed by confocal microscopy with an Eclipse Ni-E A1 (Nikon, Tokyo, Japan) microscope equipped with Plan Apo 40×/0.95 objective. To do this, PAPI was stained with the Nile Red dye prior to being dissolved in the toluene. The capsule images were taken in the transmittance and fluorescent modes. A 514 nm laser was used to excite the Nile Red fluorescence. The detection was performed in the 550–650 nm range.

The size distribution of PU capsules was studied with a Master Sizer 3000 laser diffractometer (Malvern Panalytical, Malvern, UK). The device originally measures volume-weighted size distribution, and, thus, the volume-weighted mean diameter was considered a mean capsule size. The width of capsule size distribution was characterized by Span value, which was calculated as:

(1) $$Span=\frac{Dv90-Dv10}{Dv50}$$

where *Dv*90 is the size of capsules below which 90% of the sample lies, *Dv*10 is the size of capsules below which 10% of the sample lies and *Dv*50 is the median size in the volume-weighted distribution.

The latent heat storage performance and cycling stability of the PU capsules were studied with DSC 214 Polyma (Netzsch, Selb, Germany) calorimeter in the range of −20 °C to 70 °C with a heating/cooling rate of 10 °C/min. The mass of the samples was 5 mg. Upon measurement, the collected data were processed to subtract the baseline and time-integrated to calculate the enthalpy of phase transitions in the microcapsules, employing standard data processing software.

The thermal stability of the PU capsules was studied with an STA 449 F5 Jupiter (Netzsch, Selb, Germany) device. The data were collected in the range of 30 °C to 800 °C with a heating rate of 10 °C/min. The initial mass of the samples was 6 mg. The experiments were carried out under nitrogen atmosphere.

The shape stability of NCC/PU capsules was evaluated under simulated sunlight heating. The capsules were heated with a sulfur plasma lamp, which generates a quasi-solar light with a continuous emission in the visible spectrum. The samples were placed 1 m below the lamp on the absorbing layer (a cellulose paper filter). At this distance, the light intensity was 69.5 mW/cm^2^. During the heating, the leakage of *n*-octadecane from the NCC/PU capsules was controlled by thermal imaging with a Guide D400 infrared camera (Guide Sensmart, Wuhan, China) and by visual observation. The heating was carried out for 10 min. The shape stability of the bare *n*-octadecane was evaluated in a similar way in order to compare with the stability of the encapsulated PCM. Additionally, the leakage of the loaded *n*-octadecane was evaluated by controlling the mass of the NCC/PU capsules. To do this, three portions of dried NCC/PU capsules, each of 200 mg, were put on cellulose filter papers. Then, the capsules were moved into a drying chamber with the temperature set to 60 °C. The capsules were heated for 7 h. The mass of the NCC/PU capsules was controlled every hour.

## 3. Results and Discussion

### 3.1. Synthesis of NCC/PU Capsules

**Scheme 1:**
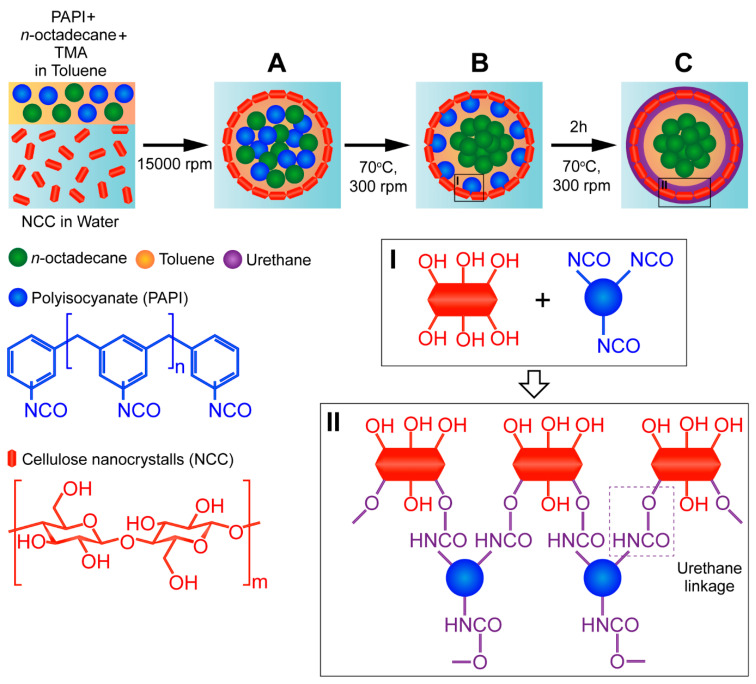
Schematic illustration of the formation of polyurethane capsules loaded with *n*-octadecane via reaction of interfacial polymerization between PAPI and NCC: (**A**) formation of oil-in-water emulsion stabilized with NCC; (**B**) initiation of the shell crosslinking in the reaction between PAPI and NCC; (**C**) formation of *n*-octadecane loaded capsules with crosslinked NCC/PU shell. (**I**) Representation of functional groups in NCC and PAPI involved into the shell crosslinking; (**II**) possible crosslinking mechanism through the formation of urethane linkages.

The NCC/PU capsules loaded with *n*-octadecane were prepared via interfacial polymerization (Scheme 1). In the first step, NCC-stabilized Pickering emulsion containing PAPI, *n*-octadecane, and TMA in the oil phase was prepared at room temperature (Scheme 1A). NCC tends to stabilize emulsion droplets due to its amphiphilic nature and intermediate wettability [29]. The(200)*β*/(220)*α* crystalline edge of cellulose chains forms a “hydrophobic face” of the NCC, which facilitates its adsorption at the oil/water interface [32,33]. Additionally, unmodified NCC has no surface groups promoting particle repulsion, which gives rise to dense NCC packing on the droplet surface. Upon emulsification, the mixture was heated to 70 °C to initiate the crosslinking between hydroxyl groups of NCC and isocyanate groups of PAPI (Scheme 1B). Heating was required to negate the steric factor and improve the reactivity of isocyanate groups in various methylene diphenyl diisocyanate isomers and higher homologues present in commercially available PAPI [26]. Additionally, heating promotes PAPI diffusion to the reaction site. In conventional interfacial polycondensation, the PU capsule wall is formed according to the moving boundary mechanism. This prompts the diffusion of polyol molecules from the aqueous phase to the oil core, where they react with isocyanate [34]. However, this is unlikely in NCC adsorbed at the oil/water interface. Apparently, in NCC/PU capsules, the PU shell was formed through the diffusion of PAPI instead. TMA is a cyclic tertiary amine with low steric hindrance which was introduced as an oil soluble catalyst to promote PU formation [35]. Finally, the reaction between isocyanates and alcohols is exothermic [Δ*H* = 24 Kcal/mol] [26]. Thus, the formation of PU linkages between NCC and PAPI was accompanied by growths in reaction mixture temperature above 70 °C. After the crosslinking was accomplished, the temperature had reduced to 70 °C. Scheme 1C shows the possible crosslinking pathways between NCC and PAPI.

**Figure 1:**
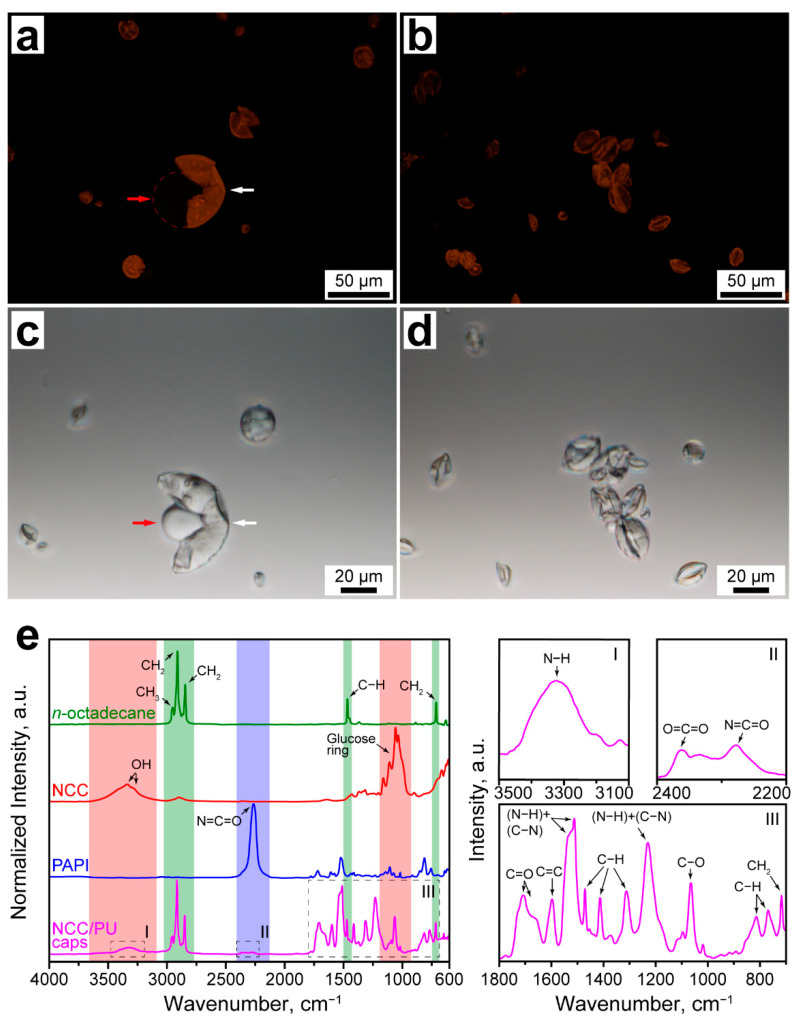
(**a**,**b**) Confocal fluorescent images of NCC/PU capsules (white and red arrows in (**a**) indicate the capsule shell and liquid core, respectively); (**c**,**d**) confocal transmission images of NCC/PU capsules (white and red arrows in (**c**) indicate the capsule shell and liquid core, respectively); (**e**) FTIR spectra of *n*-octadecane, NCC, PAPI, and NCC/PU capsules: (**I**–**III**) the insets show the specific absorption bands appeared due to PU formation.

The formation of the PU shell and encapsulation of *n*-octadecane were further studied with confocal microscopy and FTIR spectroscopy. Figure 1a–d shows the fluorescent and transmission confocal images of the NCC/PU capsules. In fluorescent images, the capsule shell formed with the Nile Red-stained PAPI can be clearly distinguished from the liquid core. Moreover, Figure 1a demonstrates that the capsule shell (marked with the white arrow) is the only source of the fluorescence. This indicates all PAPI dissolved in the oil phase (marked with the red arrow) was involved in the shell formation. Release of the liquid core from the broken capsules confirmed the successful encapsulation of the oil phase.

Figure 1e shows the comparison of the normalized FTIR absorbance spectra of *n*-octadecane, NCC, PAPI and NCC/PU capsules. The spectra of *n*-octadecane, NCC and PAPI demonstrate the distinctive maximums of absorption related to the stretching and bending of the inherent functional groups. The characteristic peaks of NCC are related to the stretching of OH groups involved in inter- and intramolecular hydrogen bonding (3335 cm^−1^ (*ν_s_*), 3292 cm^−1^ (*ν_s_*)) and the vibrations of the glucose ring (1161 cm^−1^ (*ν*_as_(C1–O–C4)), 1105 cm^−1^ (*ν*_as_(ring)), 1060 cm^−1^ (*ν*_s_(C–C)), 1035 cm^−1^ (*ν*_s_(C–OH)), 998 cm^−1^ (*ν*_s_(C–H ring))) [36]. The main inherent peak of PAPI is due to asymmetric stretching of the isocyanate group (2265 cm^−1^ (*ν*_as_(N=C=O)) [37]. The formation of polyurethane in the conventional reaction between isocyanate and alcohol implies an addition of a hydrogen atom at the N=C bond in the N=C=O group, followed by the disruption of the double bond [26]. Thus, the formation of the urethane −NHCOO− group can be detected through the formation of N−H and C−N bonds, and the absence of absorption by isocyanate and hydroxyl groups in PAPI and NCC, respectively.

The FTIR spectrum of NCC/PU capsules revealed three absorption bands resulted from the formation of urethane linkages between NCC and PAPI. The first one corresponded to the stretching vibration of the newly formed N−H bond (3323 cm^−1^ (*ν_s_*)) (Figure 1(eI)) [38]. The second one came from the stretching vibration of O=C=O group (2362 cm^−1^ (*ν_s_*)) and the residual vibration of the isocyanate group (2279 cm^−1^ (*ν*_as_(N=C=O)) (Figure 1(eII)) [38]. The intensity of absorption due to the N=C=O group was negligible, indicating almost all PAPI loaded to the oil core was involved in shell crosslinking. The appearance of a small peak at 2362 cm^−1^ suggested that PAPI may also react with water. This resulted in the generation of CO_2_ which further participated in the formation of O=C=O groups [39]. Finally, the third band demonstrated several absorption maximums associated with the formation of urethane groups: the stretching vibration of the C=O group (1703 cm^−1^ (*ν_s_*)); the framework vibrations of the C=C bond in the benzene ring (1597 cm^−1^); the double peak from the vibration of hydrogen bonded and free (N−H) + (C−N) groups (1538–1512 cm^−1^); asymmetric and symmetric bending of the C−H group (1413 cm^−1^ and 1313 cm^−1^); the bending vibration of N−H and stretching vibration of C−N groups (1230 cm^−1^); symmetric stretching vibration of the C−O−C group (1064 cm^−1^ (*ν_s_*)); out-of-plane bending vibration of the C−H group (814 cm^−1^ and 765 cm^−1^) (Figure 1(eIII)) [38]. It should be noted that the absorption peak of the C=O group was shifted to lower wavenumbers that can be associated with the formation of the large number of hydrogen bonds in the PU structure [40]. Additionally, a small shoulder appeared at 1665 cm^−1^, which may suggest the formation of urea in the −NHCONH− group due to interaction of PAPI with water [37].

Finally, the spectrum of NCC/PU capsules demonstrated absorption maximums corresponding to symmetric and antisymmetric stretching of CH_2_ groups (2915 cm^−1^ (*ν_s_*), 2849 cm^−1^ (*ν_as_*)), antisymmetric stretching of the CH_3_ group (2954 cm^−1^ (*ν_as_*)), stretching vibration of the C−H group (1470 cm^−1^ (*ν*)), and rocking vibration of the CH_2_ group (717 cm^−1^ (*ρ*)) of the paraffin chain [41].

**Figure 2:**
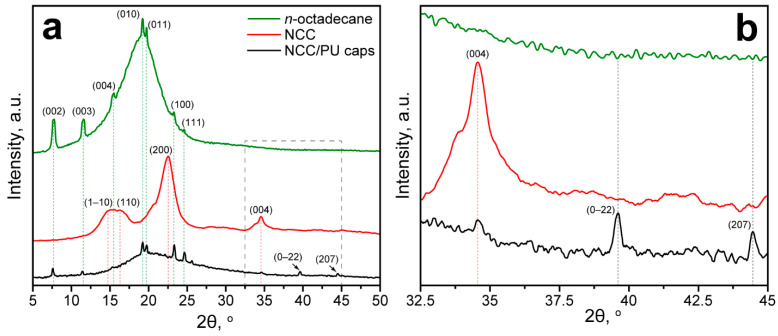
(**a**) XRD patterns of *n*-octadecane, NCC and NCC/PU capsules; (**b**) XRD patterns of *n*-octadecane, NCC and NCC/PU capsules at 2*θ* from 32.5° to 45°, demonstrating the appearance of new reflections corresponding to the *α*-form of *n*-octadecane, and reflection corresponding to NCC in the NCC/PU capsules.

The FTIR spectroscopy confirmed the formation of PU in the capsule shell. Interestingly, the FTIR spectrum of NCC/PU capsules did not reveal absorption due to vibration of the glucose ring in the NCC. Thus, the NCC/PU capsules were additionally studied with XRD analysis. Figure 2a shows the XRD patterns of initial *n*-octadecane, NCC and NCC/PU capsules. The XDR pattern of *n*-octadecane demonstrated a set of diffraction peaks at 2*θ* of 19.26°, 19.78°, 23.32°, and 24.66°, corresponding to (010), (011), (100), and (111) reflections of *β*-form (triclinic phase) of *n*-octadecane, respectively. In turn, the reflections at 2*θ* of 7.72°, 11.48°, and 15.46° are assigned to the (002), (003), and (004) planes of the *α*-form (rotator phase) of *n*-octadecane [42,43]. The XRD pattern of NCC demonstrated diffraction peaks at 2*θ* of 14.88°, 16.52°, 22.52°, and 34.62° attributed to (1–10), (110), (200), and (004) planes of cellulose I polymorph modification [44,45]. The XRD pattern of NCC/PU capsules demonstrated a set of diffraction peaks at 2*θ* similar to those of *n*-octadecane, which confirmed its successful encapsulation. Interestingly, the XRD pattern of NCC/PU capsules also revealed peaks at 2*θ* of 39.68° and 44.52°, corresponding to (0–22) and (207) planes of *α*-form of *n*-octadecane, which were not distinguishable in the XRD pattern of the initial *n*-octadecane (Figure 2b). This can be explained by the fact that the crystallization of *α*-form is driven by heterogeneous nucleation of *n*-octadecane initiated on its surface. Thus, the appearance of these peaks can be attributed to the more significant impact of the surface of the crystalline structure of encapsulated *n*-octadecane. This is a clear evidence of the formation of *n*-octadecane fraction while confined within micron-sized capsules.

Surprisingly, the XRD pattern of NCC/PU capsules did not demonstrate the main peaks related to NCC reflections. However, a broad envelope can be seen at 2*θ* from 12.5° to 32.5°. Apparently, this can be related to the lower mass content of NCC in the capsules compared with the loaded *n*-octadecane. The initial emulsion contained 3.2 g of paraffin and only 0.48 g of NCC. Thus, the NCC reflections were overlapped by the intensive reflections of n-octadecane. The presence of NCC in the capsule shell can be recognized by the reflection at 2*θ* of 34.62°, which was not overlapped by n-octadecane peaks (Figure 2b). Again, the intensity of this peak was relatively low due to the low content of NCC compared with the added PAPI (3.2 g) that was fully involved in the PU formation, as revealed by FTIR spectroscopy.

All in all, the results of confocal microscopy, FTIR spectroscopy, and XRD analysis confirmed the successful formation of NCC/PU shell through the crosslinking of NCC and PAPI via urethane linkages, along with successful encapsulation *n*-octadecane.

### 3.2. Structure of NCC/PU Capsules

**Figure 3:**
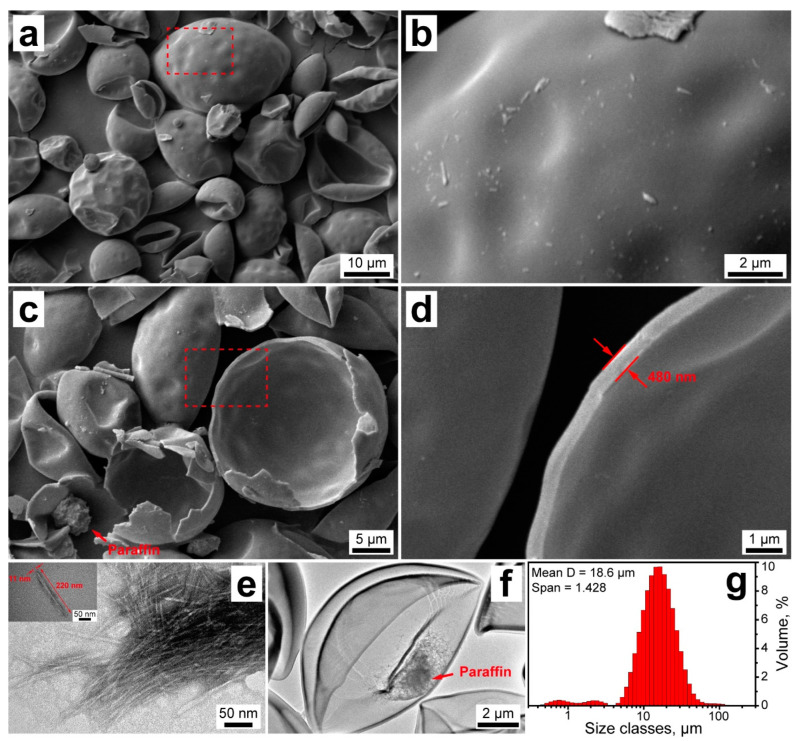
(**a**–**d**) SEM images of the NCC/PU capsules; (**e**) TEM images of the initial NCC (the inset shows the representative image of the single cellulose nanocrystal); (**f**) TEM image of the NCC/PU capsule; (**g**) size distribution of the NCC/PU capsules measured with laser diffractometry.

The morphology of the resulting NCC/PU capsules was studied with SEM (Figure 3a–d). The capsules appeared non-aggregated with mostly intact shells (Figure 3a). The capsule morphology originated from the initially formed emulsion droplets, which gave rise to their primary spherical shape. However, the capsules appeared curved inward, which can be associated with toluene evaporation after the crosslinking of the shell. Taking a closer look, the NCC/PU capsules had a smooth and uniform shell, which apparently resulted from the dense packing of NCC on the oil/water interface, and its further homogeneous crosslinking with PAPI (Figure 3b).

The SEM images also revealed a decent number of broken capsules that may be disrupted due to a high vacuum in the microscope chamber (Figure 3c). This suggests the rigid nature of NCC/PU shells. Indeed, in conventional polyurethanes, mechanical properties are defined by soft and hard domains in their structure. Elastic polyurethanes typically result from the reaction between isocyanates and polyols, with long linear polymeric chains and low functionality (two or three hydroxyl groups per molecule) [26]. The elasticity is reached through the high mobility of polymeric chains and low association of hard domains of urethane linkages via hydrogen bonding. In contrast, NCC can be considered a branched oligo-polyol with high functionality (six hydroxyl groups per monomer unit), with short chains derived from the hydroxyl groups. As a consequence of this structure, the reaction with PAPI gives a highly crosslinked polyurethane with increased rigidity, due to a high density of urethane groups which are strongly associated with hydrogen bonding [27]. The strong involvement of cellulose hydroxyl groups in the formation of urethane linkages was confirmed by FTIR spectroscopy. The spectrum of NCC/PU capsules did not demonstrate absorption peaks of the NCC hydroxyl groups after crosslinking the shell. Additionally, the paraffin core can be clearly distinguished in the SEM image of broken capsules, which confirms their core–shell structure and the successful encapsulation of *n*-octadecane.

The structure of the NCC/PU shell was studied with electron microscopy of the broken capsules (Figure 3d). According to SEM images, the shell thickness was about 450 ± 60 nm. Furthermore, at higher magnification, it can be seen that the NCC/PU shell has a single-layered structure, which suggests the formation of the interpenetrating polymeric network through the crosslinking of NCC with PAPI [46]. Apparently, the shell formation started from the reaction between NCC and PAPI at the oil/water interface. This led to the formation of an initial polyurethane layer, which was further developed into the uniform shell due to the PAPI diffusion.

Additionally, the structure of NCC and NCC/PU capsules was studied with TEM (Figure 3e,f). According to TEM, the employed NCC had a needle-like shape with a typical length of about 220 nm and a width of 10 nm (Figure 3e). This gave an aspect ratio of 22, which is relatively low for NCC [33]. Previously, it was reported that NCC with low aspect ratio is preferable for preparation of Pickering emulsions, owing to its dense organization at the oil/water interface and high coverage ratio compared with more elongated cellulose nanocrystals [47]. The TEM image of NCC/PU capsule confirmed the formation of a durable shell (Figure 3f). Furthermore, the loaded paraffin can be clearly distinguished within the capsule interior. The TEM images also demonstrated some sort of shell fracture. Apparently, this is a consequence of the shell rigidity, which had led to the minor shell cracking due to its shrinkage over the toluene evaporation. However, this minor fracturing did not exert any significant impact on the overall shell integrity and did not lead to the release of the loaded *n*-octadecane, which remained reliably sealed within the capsule.

Finally, the capsule size distribution was evaluated with laser diffraction analysis (Figure 3g). The capsules had a unimodal size distribution with the mean diameter of 18.6 µm; however, a minor fraction of particles of lower size was also detected. The Span value was 1.428, indicating a relatively narrow capsule size distribution.

### 3.3. Latent Heat Storage Performance, Thermal and Shape Stability of NCC/PU Capsules

**Figure 4:**
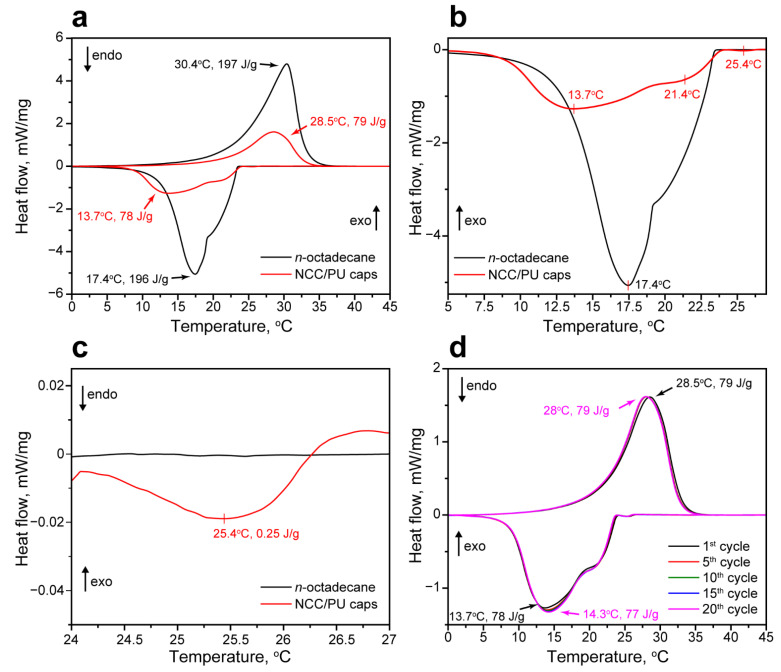
(**a**) Comparison of the melting and crystallization curves of the NCC/PU capsules and *n*-octadecane; (**b**) comparison of the crystallization curves of the NCC/PU capsules and *n*-octadecane with peak crystallization temperatures; (**c**) the exothermic peak in the crystallization curve of the encapsulated *n*-octadecane indicating the surface freezing phenomenon; (**d**) melting and crystallization curves of the NCC/PU capsules measured through the thermal reliability test.

The latent heat storage performance of the NCC/PU capsules loaded with *n*-octadecane was studied with DSC. Figure 4a shows the comparison of melting and crystallization curves of the NCC/PU capsules and bulk *n*-octadecane. *N*-octadecane had a single melting peak with a melting point *T_M_* = 30.4 °C and melting enthalpy Δ*H_M_* = 197 J/g [48]. The NCC/PU capsules demonstrated almost the same melting point *T_M_* = 28.5 °C while the melting enthalpy predictably reduced to Δ*H_M_* = 79 J/g proportionally to the loaded content of *n*-octadecane. However, the bulk and encapsulated *n*-octadecane demonstrated slightly different crystallization behavior (Figure 4b). The crystallization curve of *n*-octadecane had two distinctive segments. Previously, the XRD analysis revealed the two crystalline phases in the bulk *n*-octadecane. Thus, the first segment on the crystallization curve corresponded to the formation of the α-form or the rotator phase (R_I_) due to the heterogeneous nucleation [49]. The second segment in the crystallization curve corresponded to the formation of the *β*-form or the triclinic phase due to homogeneous nucleation, with a peak crystallization point *T_C_* = 17.4 °C. Thus, bulk *n*-octadecane showed the two types of phase transition along with the temperature decrease: the transition from isotropic liquid to rotator R_I_ phase and transition to the triclinic crystallin phase. The overall enthalpy of the bulk *n*-octadecane crystallization Δ*H_C_* was 196 J/g. The formation of the rotator phase in long-chained *n*-alkanes (starting from *C_15_*) is attributed to the surface freezing phenomenon [50]. This implies the formation of a surface crystalline monolayer at the temperatures above the bulk crystallization point. This monolayer induces the heterogeneous nucleation and formation of the bulk rotator phase [51]. Compared with the bulk *n*-octadecane, the encapsulated one had a much higher surface-to-volume ratio suggesting the surface freezing should exert a more significant impact on its crystallization. This was confirmed by a crystallization curve of NCC/PU capsules that revealed a small exothermic peak at 25.4 °C with the enthalpy of 0.25 J/g, corresponding to the surface freezing of the encapsulated *n*-octadecane (Figure 4c) [49]. The surface crystalline phase acted as a nucleation site for the heterogeneous crystallization and promoted the formation of the evident R_I_ phase with a peak crystallization temperature of 21.4 °C. The further formation of the stable triclinic phase was accompanied by a slight supercooling of 3.7 °C, compared with the bulk *n*-octadecane. This can be related to the reduced number of the nuclei that slowed down the homogeneous nucleation in encapsulated *n*-octadecane, relative to the bulk one [52]. The overall crystallization enthalpy of the encapsulated *n*-octadecane was 78 J/g; this is in agreement with its melting enthalpy.

Basing on the measured melting and crystallization enthalpies of the bulk and encapsulated *n*-octadecane, the loading efficiency *E* was calculated as:

(2) $$E=\frac{\Delta H_{M/caps}+\Delta H_{C/caps}}{\Delta H_{M/PCM}+\Delta H_{C/PCM}}\times 100\%,$$

where Δ*H_M/caps_* and Δ*H_C/caps_* are the melting and crystallization enthalpies of the encapsulated paraffin, while Δ*H_M/PCM_* and Δ*H_C/PCM_* are the melting and crystallization enthalpies of the bare paraffin. The loading efficiency demonstrates the latent heat storage and release performance of the encapsulated PCM with respect to the initial one. For the prepared NCC/PU capsules, *E* was calculated to be 40%.

The thermal storage capability *η* is another important parameter of encapsulated PCM, indicating the efficiency of thermal energy storage and release through the phase transitions in the restricted capsule volume. It can be calculated from DSC data as:

(3) $$\eta =\frac{\left(\Delta H_{M/caps}+\Delta H_{C/caps}\right)\times \Delta H_{M/PCM}}{\left(\Delta H_{M/PCM}+\Delta H_{C/PCM}\right)\times \Delta H_{M/caps}}\times 100\%$$

According to Equation (3), *η* for NCC/PU capsules was calculated to be 99%, which implies that all the encapsulated paraffin can effectively store and release latent heat. This means that the encapsulated *n*-octadecane has no confinement restrictions and is free to undergo reversible phase transitions within the capsules. This is confirmed by SEM and TEM images demonstrating plenty of free space in capsule interior, apart from the loaded *n*-octadecane which apparently comes from the evaporation of toluene.

The reliability of the latent heat storage performance of the NCC/PU capsules was studied with cyclic DSC measurements. Figure 4d shows the melting and crystallization curves of the capsules measured within 20 iterative heating/cooling cycles. In general, the DSC analysis did not reveal any substantial changes in the peak temperatures and enthalpies of the phase transitions. The only slight shift was observed after the first heating/cooling cycle; afterward, the heating and crystallization curves behaved in exactly the same way. The shift may be related to the change in the conformation and distribution of *n*-octadecane in the capsule interior through its first melting and crystallization.

**Figure 5:**
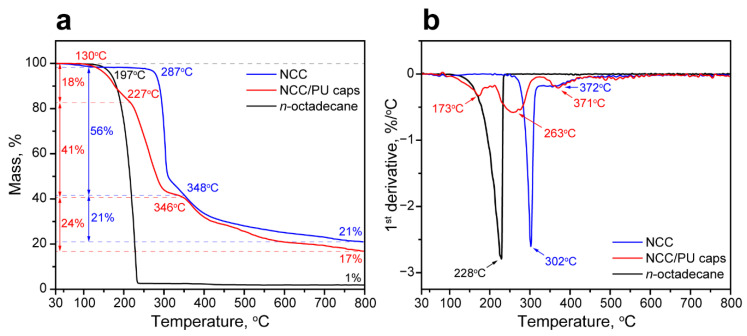
(**a**) Comparison of thermal decomposition patterns of NCC, *n*-octadecane and NCC/PU capsules; (**b**) DTGA curves of NCC, *n*-octadecane and NCC/PU capsules.

The thermal stability of the NCC/PU capsules loaded with *n*-octadecane was studied with TGA and DTGA analysis. Figure 5a,b show the comparison of TGA and DTGA curves of *n*-octadecane, NCC, and the NCC/PU capsules. The thermal decomposition of *n*-octadecane occurred in one stage, started at onset temperature *T_on_* = 197 °C with the maximum rate of decomposition temperature MRDT = 231 °C, and ended at 235 °C with the mass residue of 1% at 800 °C [53]. The decomposition of NCC involved two stages. The initial one started at *T_on_* = 287 °C with MRDT = 302 °C, and the second one started at *T_on_* = 348 °C with MRDT = 372 °C. Additionally, a minor weight loss of 2% can be seen until 100 °C, which was associated with the evaporation of bounded water. The overall weight residue at 800 °C was 21%. This decomposition pattern is inherent in the cellulose nanocrystals in cellulose I polymorph modification [54]. It should be noted that the NCC is generally produced by the acid treatment of cellulose nanofibrils. The acid dissolves the amorphous cellulose regions and leaves intact the crystalline ones. Thus, the low-temperature decomposition stage is associated with the degradation of the acid-treated NCC surface. NCC has a high surface-to-volume ratio, and, therefore, a more accessible NCC surface exerts the most prominent impact in the NCC decomposition. The following high-temperature stage is due to the degradation of the non-treated internal NCC regions. Compared with the surface, the internal regions are limited; thus, they did not have a significant impact on NCC decomposition [55].

The decomposition of the NCC/PU capsules took three stages. The first one started at *T_on_* = 150 °C with MRDT = 173 °C. This temperature range is associated with the decomposition of urethane and urea linkages in hard polyurethane segments. The hard segments tend to decompose at relatively low temperatures (as compared to the soft ones) due to their low molecular weight and association mainly through the weak hydrogen bonding [56]. The weight loss at this stage was 18%. The second stage started at *T_on_* = 227 °C with MRDT = 263 °C. This stage is due to the decomposition of the loaded *n*-octadecane accompanied by the initial decomposition of NCC. The weight loss at this stage was 41%; this corresponds to the loading efficiency of the paraffin calculated from the DSC data. Finally, the third stage started at *T_on_* = 348 °C with MRDT = 372 °C. This clearly corresponded to the high-temperature decomposition of NCC in the capsule structure. The weight loss at this stage was 24%, which also corresponds to the weight loss of 21% at the final stage of decomposition of the neat NCC. Although the NCC/PU capsules demonstrated lower overall onset decomposition temperature compared with the bare paraffin, it appeared that the decomposition of the encapsulated *n*-octadecane required higher temperatures than the pure one. Additionally, the capsules remained fairly stable below 150 °C, which is far beyond their suggested working range as derived by the melting curve. The summary of the thermal properties measured with DSC and TGA is given in Table 1.

**Figure 6:**
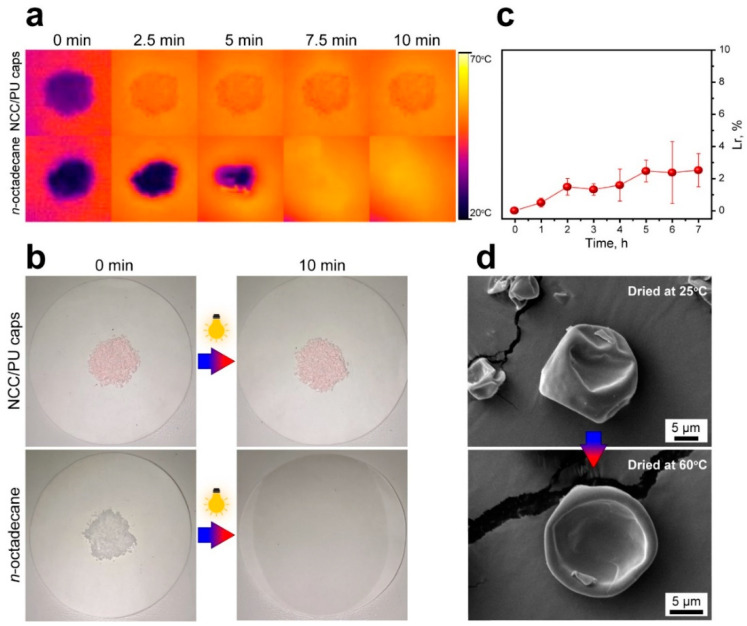
(**a**) Comparison of the shape stability of the NCC/PU capsules and bare *n*-octadecane with thermal imaging; (**b**) photos of the NCC/PU capsules and bare *n*-octadecane taken before and after heating under simulated sunlight; (**c**) the leakage rate of *n*-octadecane from the NCC/PU capsules after multiple heating cycles; (**d**) comparison of the structure of the NCC/PU capsules dried overnight at the ambient temperature (25 °C) and at 60 °C.

The shape stability of the NCC/PU capsules was studied with thermal imaging and by visual observation of heated samples, then compared with the shape stability of pure *n*-octadecane under the same conditions. To achieve a uniform surface heating, the samples were irradiated with a sulfur plasma lamp simulating a sunlight spectrum. Figure 6a shows time-lapse thermal images of the NCC/PU capsules and *n*-octadecane powder. In 10 min, the NCC/PU capsules were uniformly heated to 60 °C and no *n*-octadecane leakage was detected. This was confirmed with the capsule photos demonstrating the melting of encapsulated *n*-octadecane took place exclusively within the capsules, with no signs of leakage on the cellulose paper filter (Figure 6b). In contrast, the loss of the shape in the bare *n*-octadecane is clearly recognizable within 2.5 min of heating. The photos demonstrated the complete melting of *n*-octadecane, and its spreading and absorption by the filter in 10 min under the lamp.

For further consideration of the possible leakage issue of the encapsulated *n*-octadecane, the material leakage rate (*Lr*) was measured. *Lr* was calculated as

(4) $$Lr=\frac{m_{0}-m_{t}}{m_{0}}\times 100\%,$$

where *m*_0_ is the initial mass of the NCC/PU capsules, and *m_t_* is the mass measured at a prescribed time interval. The result is given in Figure 6c. In 7 h of heating at 60 °C, the NCC/PU capsules demonstrated a leakage rate of about 2.5%, indicating the high reliability of the NCC/PU shell. The measured *Lr* is comparable to the one reported for the capsules prepared by stabilization of emulsion droplets with graphene sheets and further polymerization of melamine–formaldehyde shell [57]. This confirms the feasibility of the proposed approach for the preparation of capsule shells with high protective properties via polymerization at the oil/water interface of Pickering emulsions.

Finally, the structure of the heated and non-heated capsules was compared using SEM. Figure 6d shows the SEM images of NCC/PU capsules dried at the ambient temperature (25 °C) and in the drying box at 60 °C overnight. It can be seen that the overnight heating to 60 °C did not affect the shell structure at the microscale and the capsules were robust to changes in temperature.

## 4. Conclusions

Shape-stable capsules with latent heat storage properties were successfully prepared via the interfacial polymerization of Pickering emulsion stabilized with cellulose nanocrystals. The stable capsule shell was achieved by the crosslinking of NCC with urethane linkages through the reaction between cellulose hydroxyl groups and isocyanate groups of PAPI dissolved in the oil phase. In this direct crosslinking, cellulose molecules acted as a branched oligo-polyol with high functionality. This gave rise to a rigid and highly crosslinked polyurethane structure due to the high density of urethane groups. The involvement of cellulose hydroxyl groups in the formation of polyurethane was confirmed by FTIR spectroscopy, while the rigid nature of the resulting NCC/PU shell was demonstrated by electron microscopy. The successful formation of the NCC/PU shell and encapsulation of *n*-octadecane was also confirmed by fluorescent confocal microscopy and XRD analysis. Additionally, the electron microscopy revealed the resulting capsules had a quasi-spherical shape with a uniform and smooth shell and a thickness of 450 ± 60 nm. The mean capsule size was 18.6 µm.

DSC demonstrated that the resulting NCC/PU capsules had a latent heat storage capacity of 79 J/g with 40% loading efficiency of *n*-octadecane. The capsules demonstrated good reliability of latent heat storage properties during at least 20 heating/cooling cycles. The TGA analysis revealed the encapsulation of *n*-octadecane in the NCC/PU capsules improved its thermal stability and shifted its decomposition to higher temperatures. Additionally, TGA confirmed the capsules remained stable until the initiation of decomposition of urethane linkages at 150 °C. The thermal imaging demonstrated that the NCC/PU shells greatly improved the shape stability of the loaded *n*-octadecane and effectively prevented its leakage under heating with simulated sunlight.

The future studies may be devoted to the improvement of the latent heat storage properties of the capsules, through the optimization of paraffin loading and the ratio of aqueous to oil phase in the initial Pickering emulsion. As far as real practical application is concerned, the development of nanosized capsules is highly desirable due to their increased heat transfer area and their suitability for dense packing in thin material layers [58]. A single-step method of shell crosslinking and mass production of the capsules are also of particular interest.

## Figures and Tables

**Table 1:** Summary of thermal properties of *n*-octadecane, NCC, and the NCC/PU capsules measured with DSC and TGA.

Sample	DSC					TGA/DTGA			
	*T_M_*, °C	Δ*H_M_*, J/g	*T_C_*, °C	Δ*H_C_*, J/g	*E*, %	*T_on_*, °C *	MRDT, °C *	Weight Loss, % *	Residue at 800 °C, % *
*n*-octadecane	30.4	197	17.4	196	-	197	228	99	1
PU/NCC capsules	28.5	79	13.7	78	40	150, 227, 346	173, 263, 371	18, 41, 24	17
NCC	-	-	-	-	-	287, 348	302, 372	56, 21	21

* Multiple values indicate different decomposition stages.

## Data Availability

The data that support the findings of this study are available from the corresponding author upon reasonable request.
